# Complete Heart Block After Aortic Valve Repair and Septal Myectomy in a Patient With Rheumatic Heart Disease

**DOI:** 10.7759/cureus.53347

**Published:** 2024-01-31

**Authors:** Marvi Moreno, Wilbur Ji, Brianna Yee, Kachon Lei, Chowdhury Ahsan

**Affiliations:** 1 Internal Medicine, Kirk Kerkorian School of Medicine at University of Nevada, Las Vegas (UNLV), Las Vegas, USA; 2 Internal Medicine, University of California, San Francisco, San Francisco, USA; 3 Cardiology, Kirk Kerkorian School of Medicine at University of Nevada, Las Vegas (UNLV), Las Vegas, USA; 4 Internal Medicine and Cardiology, Kirk Kerkorian School of Medicine at University of Nevada, Las Vegas (UNLV), Las Vegas, USA

**Keywords:** preoperative assessment, complete heart block, rheumatic heart disease, aortic valve repair, septal myectomy, hypertrophic obstructive cardiomyopathy

## Abstract

Surgical myectomy with concomitant valvular repair has been demonstrated to be safe in the treatment of hypertrophic obstructive cardiomyopathy (HOCM). It is unclear which risk factors predispose patients to develop complete heart block (CHB). We present a unique case of a 66-year-old female with rheumatic heart disease and HOCM admitted for aortic valve (AV) repair and septal myectomy, complicated by a presentation of complete heart block. The histology slide showed fibrosis of the septum, suggesting atrioventricular conduction disease from rheumatic fever, which likely contributed to her presentation. This case highlights the importance of elucidating the etiology of HOCM before undergoing cardiac surgery to guide postsurgical management and improve clinical outcomes.

## Introduction

Hypertrophic cardiomyopathy affects 1 in 500 people in the United States [1]. The etiology of hypertrophic obstructive cardiomyopathy (HOCM) includes genetic mutation, infiltrative cardiomyopathy, and chronic hypertension. Comparatively, HOCM caused by rheumatic heart disease is rare. Alcohol septal ablation is considered to be the gold standard in the treatment of isolated septal HOCM. However, surgical management is warranted in symptomatic patients presenting with concomitant valvular abnormalities. Immediate postoperative complete heart block (CHB) is rare, occurring in <3% of patients, which is further decreased in patients who have an underlying normal conduction system [2]. No data has been reported regarding delayed presentations of CHB following surgical myectomy, though the incidence was consistent at <3% when evaluating patients up to 30 days postoperatively [3]. Herein, we report a case of delayed CHB after surgical septal myectomy and aortic valve (AV) repair in a patient with a history of rheumatic heart disease and a normal preoperative electrocardiogram.

Our case highlights the importance of early risk stratification in patients with HOCM presenting for elective septal myectomy. Notably, the etiology of HOCM should be elucidated before surgery. A history of rheumatic heart disease as well as conduction system disease should prompt providers to consider further workup to aid in the decision of implementing prolonged temporary epicardial or permanent pacing in HOCM patients undergoing surgical septal myectomy to avoid postoperative mortality.

## Case presentation

**Figure 1:**
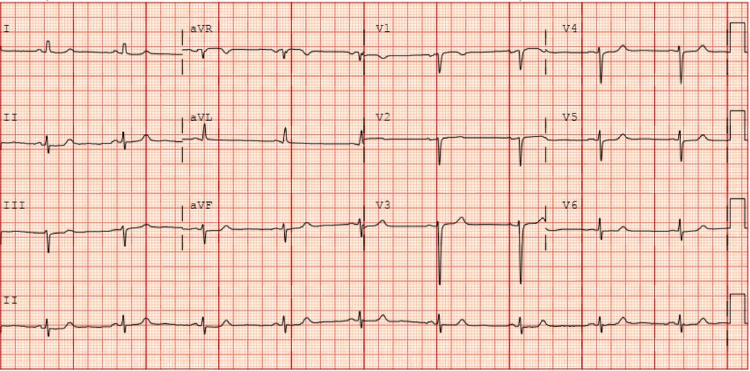
Preoperative EKG showing normal sinus rhythm There is no evidence of conduction disease, heart block, or significant ischemic changes on the EKG.

**Figure 2:**
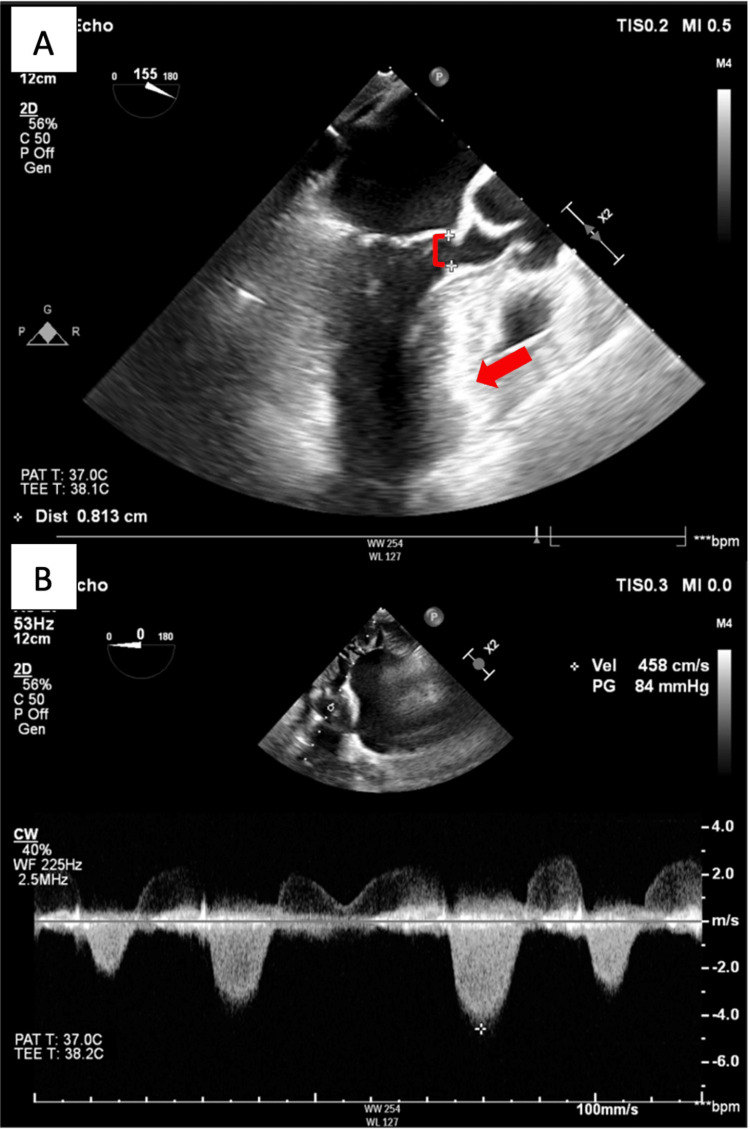
Intraoperative TEE demonstrating significant septal bulge and transvalvular systolic gradient across the AV A: Septal bulge (red arrow) with reduced systolic LVOT diameter (red bracket) at 0.81 cm (normal >2 cm);  B: Continuous wave Doppler across AV during systole. Subvalvular LVOT gradient at 84 mmHg (normal <30 mmHg) consistent with LVOT obstruction in the setting of septal HOCM. The interventricular septum diameter was 2 cm (not measured on the image). TEE: Transesophageal echocardiogram, LVOT: Left ventricular outflow tract, AV: Aortic valve, HOCM: Hypertrophic obstructive cardiomyopathy

A 66-year-old female with a past medical history of rheumatic heart disease, thyroid cancer status post-thyroidectomy, and chronic kidney disease presented to the clinic with reported shortness of breath. She was found to have moderate central aortic insufficiency caused by restricted leaflet motion of the AV, as well as left ventricular outflow tract (LVOT) obstruction secondary to septal hypertrophy (with an interventricular septal diameter of 2.0 cm) on a transthoracic echocardiogram (TTE). An EKG showed normal sinus rhythm, no significant ST-T changes, and no concern for conduction abnormalities (Figure 1). A subsequent transesophageal echocardiogram (TEE) before surgery demonstrated a calcific and restricted AV with an LVOT pressure gradient of 84 mmHg and a preserved ejection fraction (Figure 2). A preoperative left-heart catheterization revealed non-obstructive coronary artery disease.

The patient arrived at the hospital for elective cardiovascular surgery for septal myectomy. Given the concomitant aortic insufficiency and restricted AV, she was also planned to undergo surgical AV repair. Initial vital signs were unremarkable, with a temperature of 97.5F, a heart rate of 68 bpm, a respiratory rate of 17 bpm, and a blood pressure of 148/98 mmHg. She was taken to the surgical suite, where she underwent surgical septal myectomy and repair of the AV with left atrial appendage closure. The LVOT diameter improved from 0.7 cm to 1.8 cm, and the gradient was reduced from 84 mmHg to 18 mmHg. Epicardial wires and chest tubes were removed on postoperative day (POD) 2.

**Figure 3:**
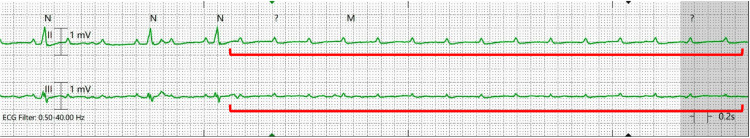
Leads 2 and 3 on POD 2 telemetry demonstrating CHB developing into asystole (red brackets) and cardiac arrest POD: Postoperative day, CHB: Complete heart block

On POD 4, the patient witnessed a loss of consciousness after ambulation and became unresponsive. Her telemetry monitor showed asystole/CHB (Figure 3). A code blue was activated, and return of spontaneous circulation (ROSC) was achieved after one round of epinephrine and advanced cardiac life support (ACLS). During an evaluation of bedside ultrasound, the patient became unresponsive again, with the ultrasound visualizing the progression of bradycardia to asystole. Advanced cardiac life support was re-initiated, the patient was intubated, and an epinephrine drip was administered after achieving ROSC. A temporary transvenous pacemaker was promptly placed. Laboratory findings during the code revealed a normal CBC and electrolyte panel. The patient improved and was successfully extubated on POD 5.

**Figure 4:**
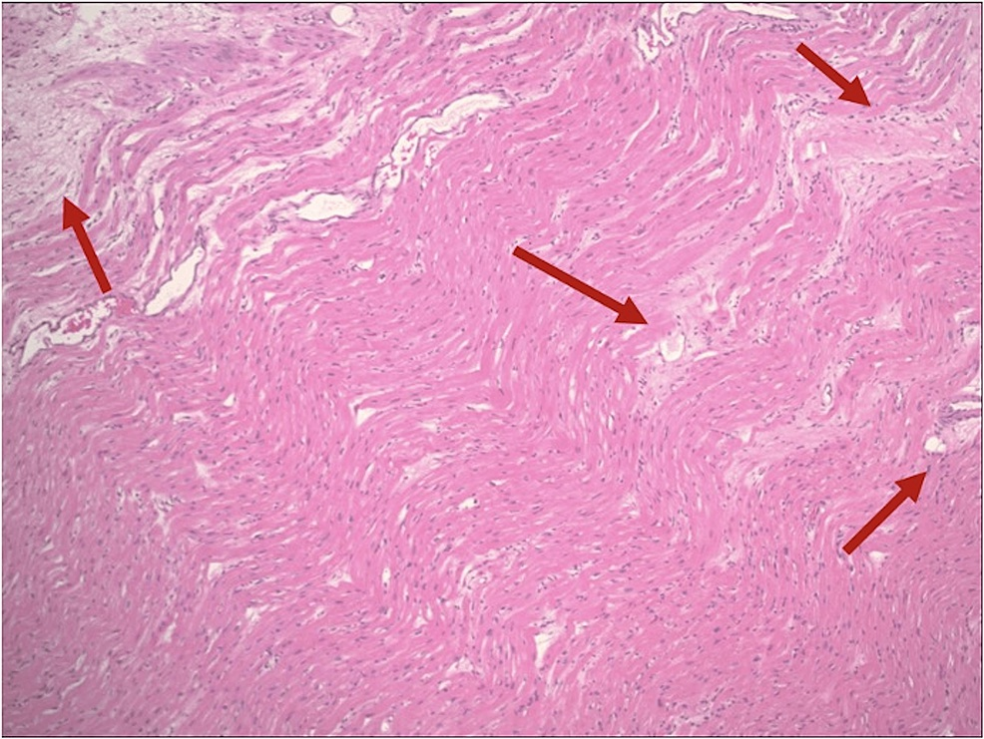
Hematoxylin and eosin stain of the myocardial septum after surgery The histopathological examination revealed focal scarring with neutrophilic infiltration (Aschoff bodies indicated by the red arrows), consistent with the history of rheumatic heart disease, with no overt evidence of hypertrophic cardiomyopathy. Light pink areas indicate fibrosis, dark pink regions suggest cardiomyocytes, and the blue specks mark the nuclei.

Histopathology slides from the operation resulted later in the hospital course, showing focal scarring with neutrophilic infiltration of the atrioventricular node in the septum, consistent with a history of rheumatic heart disease (Figure 4). She underwent implantation of a permanent dual-chamber pacemaker, and she was successfully discharged to a rehab facility.

## Discussion

The management of HOCM is clinically challenging in the setting of other cardiac comorbidities [1,4]. Hypertrophic obstructive cardiomyopathy caused by rheumatic heart disease is rare. According to the current guidelines, cardiac MRI (CMRI) and TTE are the gold standards for diagnosing and risk-stratifying patients with HOCM [1]. An interventricular septal diameter of 2.0 cm indicates septal hypertrophy and potential LVOT obstruction (when the gradient is above 30 mmHg), leading to symptoms such as dyspnea, fatigue, and chest pain. Septal myectomy is the recommended surgical treatment to alleviate obstruction when medical therapy is inadequate [1,2]. However, there is a lack of data on how to minimize postoperative conduction complications in alcohol septal ablation and surgical myectomy.

Current literature displays a range of CHB incidence, from as little as 0.6% to <5% of patients when evaluating CHB 30 days after surgery [1,3,5]. In addition, a large retrospective study on patient outcomes with septal HOCM who underwent either alcohol septal ablation or surgical myectomy found a positive correlation between pre-existing conduction disease (right bundle branch or left bundle branch block) on EKG and long-term postoperative mortality [5]. Complete heart block developed in 2.3% of all post-myectomy patients (worse with pre-existing right bundle branch block), which was significantly less in patients with a normal conduction system [5]. With technological advancements in surgical techniques, only 0.4% of patients with baseline normal conduction developed CHB [5]. 

Rheumatic heart disease is a well-recognized cause of atrioventricular conduction disease, especially in cases of severe carditis. Diffuse inflammatory involvement of the myocardium leads to scarring and conduction abnormalities. This may lead to varying degrees of atrioventricular block [6]. In the setting of HOCM and valvular diseases, surgical myectomy with concurrent valve surgery was demonstrated to be safe. If surgical myectomy were to be pursued, other left-sided valvular pathology detected through TTE would warrant simultaneous repair or replacement, according to the 2020 American College of Cardiology (ACC) guideline [1,3]. 

In our patient’s case, the decision was made to repair the AV at the same time as the septal myectomy. Unfortunately, the AV repair, septal myectomy, and an underlying diseased atrioventricular node all contributed to the development of CHB, leading to her cardiac arrest. Our patient, who had a normal EKG before her surgery, suggested a baseline normal conduction system. Yet, her hospital course following myectomy was complicated by CHB. Her cardiac arrest cannot be explained by coronary artery disease, given her negative coronary angiogram. In addition, this rare complication raises suspicion for other predisposing factors.

Interestingly, her pathology slides showed significant focal scarring of the atrioventricular node in the septum (Aschoff bodies), likely secondary to her history of rheumatic heart disease. In addition, there was evidence of restricted leaflet motion of the AV and scarring or thinning of the left and right coronary cusps. These serve as evidence that there might be a significant history of carditis in this individual, and her history of rheumatic heart disease played a significant role in her development of CHB. Considering her prior medical history as a form of risk stratification, a low threshold for prolonged transvenous or epicardial pacing would serve as a protective mechanism for our patient.

As an additional tool, retrospective studies suggest possible benefits of using CMRI to risk-stratify HOCM patients before intervention [7-9]. Cardiac MRI helps identify the location and amount of scar tissue within the heart. Late gadolinium enhancement of >15% in the left ventricle is associated with perioperative arrhythmias, especially if found within the atrioventricular node [9]. This may help guide clinicians on the duration of temporary perioperative pacing support, as prolonged transvenous/epicardial pacing in patients with a high risk of intrinsic scar tissue and conduction abnormalities may reduce inpatient mortality. Preoperative CMRI is performed in 63.2% of patients [3]. However, we emphasize the utilization of preoperative CMRI before surgical myectomy for all HOCM patients, which may help risk-stratify patients for their need for postoperative pacing. Our patient, with baseline normal cardiac conduction and low suspicion for acute conduction complications, would have benefited from preoperative imaging to reduce the risk of perioperative arrhythmias further and to help guide the duration of postoperative pacing support.

## Conclusions

Our case highlights a rare cause of septal HOCM caused by rheumatic heart disease. Increased suspicion for underlying scar tissue in these patients, regardless of apparent intact conduction systems by EKG, will help guide perioperative management. Once appropriate risk stratification is achieved, the decision for prolonged pacing following surgical intervention should be considered to prevent postoperative mortality, particularly in patients with a history of rheumatic heart disease.
